# Bilirubin-associated single nucleotide polymorphism (SNP) and respiratory health outcomes: a mendelian randomization study

**DOI:** 10.1186/s12931-023-02471-w

**Published:** 2023-07-20

**Authors:** Arianne K. Baldomero, David M. MacDonald, Adam Kaplan, Eric Lock, Michael H. Cho, Russell Bowler, Lucas Gillenwater, Ken M. Kunisaki, Chris H. Wendt

**Affiliations:** 1 Pulmonary, Allergy, Critical Care, and Sleep Medicine, Minneapolis Veterans Affairs Health Care System, One Veterans Drive, Minneapolis, MN 55417 USA; 2 Pulmonary, Allergy, Critical Care, and Sleep Medicine, University of Minnesota, Minneapolis, MN USA; 3 Division of Biostatistics, School of Public Health, University of Minnesota, Minneapolis, MN USA; 4 Division of Pulmonary and Critical Care, Department of Medicine, Brigham and Women’s Hospital, Harvard Medical School, Boston, MA USA; 5 Department of Medicine, National Jewish Health, Denver, CO USA; 6 Computational Bioscience Program, University of Colorado Anschutz Medical Campus, Aurora, CO USA

**Keywords:** Pulmonary disease, chronic obstructive, Bilirubin, Antioxidants, Genetic association studies

## Abstract

**Background:**

Observational studies have shown an association between higher bilirubin levels and improved respiratory health outcomes. Targeting higher bilirubin levels has been proposed as a novel therapeutic strategy in COPD. However, bilirubin levels are influenced by multiple intrinsic and extrinsic factors, and these observational studies are prone to confounding. Genetic analyses are one approach to overcoming residual confounding in observational studies.

**Objectives:**

To test associations between a genetic determinant of bilirubin levels and respiratory health outcomes.

**Methods:**

COPDGene participants underwent genotyping at the baseline visit. We confirmed established associations between homozygosity for rs6742078 and higher bilirubin, and between higher bilirubin and decreased risk of acute respiratory events within this cohort. For our primary analysis, we used negative binomial regression to test associations between homozygosity for rs6742078 and rate of acute respiratory events.

**Results:**

8,727 participants (n = 6,228 non-Hispanic white and 2,499 African American) were included. Higher bilirubin was associated with decreased rate of acute respiratory events [incidence rate ratio (IRR) 0.85, 95% CI 0.75 to 0.96 per SD increase in bilirubin intensity]. We did not find significant associations between homozygosity for rs6742078 and acute respiratory events (IRR 0.94, 95% CI 0.70 to 1.25 for non-Hispanic white and 1.09, 95% CI 0.91 to 1.31 for African American participants).

**Conclusions:**

A genetic determinant of higher bilirubin levels was not associated with better respiratory health outcomes. These results do not support targeting higher bilirubin levels as a therapeutic strategy in COPD.

**Supplementary Information:**

The online version contains supplementary material available at 10.1186/s12931-023-02471-w.

## Introduction

Chronic obstructive pulmonary disease (COPD) is a common respiratory disorder that is among the leading causes of death worldwide. [1] Much of the morbidity, mortality, and economic costs that result from COPD are a result of acute exacerbations of COPD (AECOPD), which are characterized by periods of increased respiratory symptoms requiring a change in treatment. [2–4] Current therapies for COPD are only modestly effective at reducing AECOPD, and new approaches are needed to reduce the devastating impact of this common disease.

Oxidative stress is increased in COPD, and further increases during AECOPD. [5] Oxidative stress has been implicated in COPD and AECOPD pathogenesis and is a potential therapeutic target. Bilirubin, the main metabolic end-product of heme degradation, is a potent anti-oxidant that scavenges peroxyl radicals and inhibits membrane-bound nicotinamide adenine dinucleotide phosphate (NADPH) oxidase, which is a large intracellular source of reactive oxygen species. [6,7] We and others have demonstrated in large observational studies that higher serum bilirubin concentrations are associated with lower risk of AECOPD [8,9], slower rate of lung function decline [10,11], and lower all-cause mortality. [12] In a recent systematic review we found that higher serum bilirubin concentrations may be associated with decreased mortality, lower risk of AECOPD, reduced incidence of COPD diagnosis, and improved lung function. [13] Although these data suggest protective effects of serum bilirubin levels, and thus that manipulation of bilirubin levels may be a therapeutic target in COPD, these observational study designs are prone to biases, confounding, and reverse causation. Mendelian randomization studies are one way of overcoming some of the problems inherent in observational studies. [14] Mendelian randomization leverages genetic variants that affect a risk factor (e.g. a variant that leads to higher bilirubin levels) to test if an observational association is consistent with a causal effect.

Serum bilirubin concentrations are heritable, with the most observed example being Gilbert’s disease, a benign condition associated with increased plasma concentrations of unconjugated bilirubin. Decreased UDP-glucuronosyltransferase 1A1 (UGT1A1) enzyme activity leads to the unconjugated hyperbilirubinemia seen in Gilbert’s disease. [15] The UGT1A1 gene codes for the hepatic enzyme responsible for bilirubin glucuronidation which is an essential step in biliary excretion of bilirubin. UGT1A1 is the major genetic determinant of serum bilirubin, and in a genome wide association study (GWAS) meta-analysis the UGT1A1 single nucleotide polymorphism (SNP) rs6742078 explained 16.7 to 18.1% of the variation in circulating bilirubin levels (while other SNPs explained only 0.5 to 0.6% of the variation). . [16] GWAS have analyzed associations between SNPs and respiratory health outcomes, but do so blindly and may miss important, hypothesis driven associations that do not meet the statistical power required in a GWAS. [17] One previous analysis showed an association between rs6742078 and less obstruction on pulmonary function testing, [18] but we are aware of no analyses that have evaluated the association between rs6742078 (or other genetic determinants of bilirubin levels) and AECOPD or longitudinal measures of respiratory health.

We used data from the Genetic Epidemiology of COPD (COPDGene) study [19] to evaluate if the UGT1A1 SNP rs6742078 is associated with acute respiratory events, including AECOPD, and in secondary analysis whether it is associated with other respiratory health outcomes.

## Methods

### Participants

A full description of COPDGene has been previously published. [19] Briefly, COPDGene is a multi-center, prospective, longitudinal cohort study of current and former smokers, with and without COPD. COPDGene enrolled more than 10,000 non-Hispanic white and African American participants between 2008 and 2011. Participants with known alpha-1-antitrypsin deficiency were excluded, and participants underwent genotyping using the Illumina Omni-Express Chip at the baseline visit. In this study we included all participants who had baseline data available for genotype, race, age, sex, and pack years of tobacco smoking.

### Procedures, definitions, and selection of SNPs

Respiratory exacerbations in COPDGene are defined as an increase in respiratory symptoms that require systemic steroids and/or antibiotics. Severe exacerbations are defined as those that required an emergency department visit or hospitalization. Exacerbation data were collected longitudinally in the COPDGene Longitudinal Follow-up program through automated telephone calls and web-based questions every 3–6 months. [20] In this analysis we use the term ‘acute respiratory event’ as opposed to AECOPD, as not all participants had COPD by spirometry criteria.

Serum bilirubin levels were not measured in the COPDGene protocol, but bilirubin intensity by mass spectrometry was available from a subset of participants who had serum metabolite profiling. [21,22].

SNP rs6742078 was selected based upon previously published associations with serum bilirubin levels and respiratory health outcomes. [16,18] Three other SNPs (rs4148324, rs4148325, and rs887829) explained a similar percentage (~ 18%) of the variation in bilirubin levels in a genome wide association study (GWAS) and we analyzed these SNPs as well. [16].

### Statistical analysis

We tested the correlation of bilirubin intensities to SNP copies. Bilirubin intensities (n = 1,028) were centered to have mean 0 and scaled to have standard deviation 1 (see e-Fig. 1 for histogram). Bilirubin intensities were then compared between participants who had 0, 1, or 2 copies for each of the 4 SNPs of interest using ANOVA F-tests. All 4 SNPs showed a similar and highly significant (p-value < 0.0001) relationship between SNP count (0, 1, or 2) and bilirubin levels. For each SNP there were significantly higher abundances of bilirubin observed for those with 2 copies of the SNP when compared with those with 0 or 1 copies (e-Fig. 2). Additionally, there were no significant differences between those with 0 or 1 copy. Correlations between SNPs were then measured using the Pearson correlation for each SNP pair. The 4 SNPs of interest were highly correlated with correlation coefficients > 0.90 (e-Fig. 3). Due to these high correlations, we focused only on the original SNP of interest, rs6742078. Given the findings comparing bilirubin intensities by number of SNP copies, we dichotomized the cohort into those homozygous for the minor allele and all other genotypes before testing associations with respiratory health outcomes.

In our primary analysis we used zero-inflated negative binomial regression to test the association between homozygosity for SNP rs6742078 and the annualized rate of acute respiratory events. All models were adjusted for age, sex, pack-years of smoking history, and principal components of genetic ancestry. African American and non-Hispanic white participants were analyzed separately to incorporate principal components of genetic ancestry. We also tested associations between SNP rs6742078 and secondary outcomes of mortality, forced expiratory volume in 1-second (FEV_1_), forced vital capacity (FVC), FEV_1_/FVC ratio, and longitudinal changes in FEV_1_, FVC, emphysema, gas trapping, airway wall area, Pi10 (the square-root of the wall area of a hypothetical 10 mm internal parameter airway, another marker of airway wall checkness 23), airway wall thickness, 6-minute walk distance, modified Medical Research Council (mMRC) dyspnea scale score, and St. George’s Respiratory Questionnaire (SGRQ) total score. For analyses, we discretized the 5-year change in mMRC into whether the participants had an increase (strictly positive difference) versus no change or a decrease. For mortality and mMRC as binary outcomes we employed logistic regression. We also analyzed mortality as a survival outcome using Cox proportional hazards regression. All other models used linear regression. Secondary outcome models adjusted for genetic ancestry, sex, age and pack-years of smoking history. All-cause mortality was also adjusted for clinical site at the first time point, and when allowed, months of follow-up was used as an offset in the regression models.

In an analysis limited to participants who had bilirubin intensities available, we used normalized bilirubin levels as the primary predictor and performed the same analyses. For the primary outcome, we also performed an analysis stratified by sex to test whether relationships varied between males and females.

## Results

The primary analysis of long-term follow-up exacerbation rates included 8648 participants. Six hundred and fifty-eight participants were excluded for missing genetic sequencing, 1326 for missing long-term follow-up measures, 77 for missing genetic ancestry principal components, 8 for missing rs6742078 values, and 3 for missing pack years of tobacco smoking; full data was observed for race, age, and gender. There were 2498 African American and 6150 non-Hispanic white participants. Mean (standard deviation) follow up was 5.6 (0.9) years (maximum 9 years) and there were a total of 20,380 acute respiratory events.

Baseline characteristics stratified by non-Hispanic white and African American race, and by SNP count (0–1 and 2) are shown in Table 1. Non-Hispanic white participants were older than African American participants. The percentage of female participants and body mass index (BMI) were similar. African American participants were more likely to be current smokers but had less pack-years of smoking history. Non-Hispanic white participants had worse lung function as measured by FEV_1_/FVC ratio and FEV_1_ percent predicted and had more emphysema and air trapping on quantitative CT measurement. The baseline characteristics by SNP count were similar in each group.

**Table 1:** Baseline characteristics by bilirubin SNP rs6742078 alleles. Results are reported as mean (standard deviation) unless otherwise noted

Genotype	Non-Hispanic White			African American		
	0–1 copies of rs6742078	2 copies of rs6742078	Total	0–1 copies of rs6742078	2 copies of rs6742078	Total
	n = 5572	n = 656	n = 6228	n = 2096	n = 403	n = 2499
Age, years	62.52 (8.71)	62.33 (8.52)	62.50 (8.69)	55.26 (7.54)	54.32 (6.92)	55.11 (7.45)
Female, n (%)	2696 (48.4)	331 (50.5)	3027 (48.6)	1013 (48.3)	184 (45.7)	1197 (47.9)
BMI, kg/m^2^	28.82 (6.07)	28.46 (5.84)	28.79 (6.04)	29.44 (6.90)	29.48 (6.91)	29.45 (6.90)
**Smoking History**						
Current smoker, n (%)	2031 (36.5)	237 (36.1)	2268 (36.4)	1621 (77.3)	315 (78.2)	1936 (77.5)
Pack-years	47.11 (25.96)	46.57 (24.71)	47.05 (25.83)	38.17 (21.35)	38.24 (20.85)	38.18 (21.27)
**Spirometry** ^[1]^						
FEV_1_/FVC ratio	0.64 (0.16)	0.64 (0.17)	0.64 (0.17)	0.71 (0.14)	0.72 (0.14)	0.71 (0.14)
FEV_1_% predicted	73.68 (25.60)	73.53 (26.56)	73.67 (25.70)	80.97 (24.38)	79.89 (23.64)	80.80 (24.26)
FVC % predicted	85.89 (17.85)	86.19 (17.95)	85.92 (17.86)	88.90 (18.96)	86.54 (17.92)	88.52 (18.82)
Normal spirometry, n (%)	2112 (38.0)	259 (39.7)	2371 (38.2)	1084 (52.2)	207 (52.4)	1291 (52.2)
GOLD Stage 1–2, n (%)	1699 (30.6)	193 (29.6)	1892 (30.5)	418 (20.1)	75 (19.0)	493 (19.9)
GOLD Stage 3–4, n (%)	1144 (20.6)	136 (20.8)	1280 (20.6)	241 (11.6)	50 (12.7)	291 (11.8)
PRISm, n (%)	601 (10.8)	65 (10.0)	666 (10.7)	334 (16.1)	63 (15.9)	397 (16.1)
**Quantitative CT Chest Measurements**						
AWT-Pi10, mm	2.33 (0.61)	2.31 (0.60)	2.33 (0.60)	2.37 (0.61)	2.36 (0.62)	2.37 (0.61)
Airway wall area, %	50.81 (8.35)	50.54 (8.34)	50.78 (8.35)	50.99 (8.74)	51.05 (8.78)	51.00 (8.74)
Emphysema, %	7.55 (10.40)	7.96 (10.51)	7.60 (10.41)	4.17 (8.16)	3.62 (7.36)	4.08 (8.04)
Air trapping, %	23.85 (20.08)	24.81 (20.62)	23.95 (20.14)	16.45 (17.64)	14.67 (16.95)	16.17 (17.54)
**Clinical characteristics**						
SGRQ, total score	25.8 (22.2)	26.39 (22.90)	25.85 (22.29)	29.84 (23.38)	28.27 (22.89)	29.59 (23.31)
MMRC > 2, n (%)	1447 (26.0)	178 (27.2)	1625 (26.1)	725 (34.6)	123 (30.5)	848 (34.0)
6-MWD, feet	1400 (389)	1422 (386)	1402 (389)	1243 (388)	1274 (363)	1249 (384)
**Comorbidities**						
Cong. Heart Failure, n (%)	170 (3.1)	23 (3.5)	193 (3.1)	74 (3.5)	11 (2.7)	85 (3.4)
Coronary Art. Dis., n (%)	496 (8.9)	59 (9.0)	555 (8.9)	56 (2.7)	13 (3.2)	69 (2.8)
Diabetes, n (%)	680 (12.2)	72 (11.0)	752 (12.1)	331 (15.8)	67 (16.6)	398 (15.9)
Hypertension, n (%)	2395 (43.0)	261 (39.8)	2656 (42.7)	997 (47.6)	181 (44.9)	1178 (47.1)
Hyperlipidemia, n (%)	2589 (46.5)	302 (46.0)	2891 (46.4)	583 (27.8)	108 (26.8)	691 (27.7)
Asthma, n (%)	933 (16.7)	115 (17.5)	1048 (16.8)	514 (24.5)	102 (25.3)	616 (24.6)

6-MWD, 6-minute walk test distance; AWT, airway wall thickness; AWT-Pi10, square root of the wall area for a theoretical airway with an internal perimeter of 10 mm; BMI, body mass index; FEV_1_, forced expiratory volume in 1-second; FVC, forced vital capacity; GOLD, Global Initiative for Chronic Obstructive Lung Disease; mm, millimeters; MMRC, Modified Medical Research Council Dyspnea Scale; PRISm, preserved ratio impaired spirometry; SGRQ, St. George’s Respiratory Questionnaire

1. Normal spirometry was defined as FEV_1_/FVC ratio ≥ 0.7 and FEV_1_ ≥ 80% predicted; GOLD Stage 1–2 as FEV_1_/FVC ratio < 0.7 and FEV_1_ > 50% predicted; GOLD Stage 3–4 as FEV_1_/FVC ratio < 0.7 and FEV_1_ ≤ 50% predicted; and PRISm as FEV_1_/FVC ratio ≥ 0.7 and FEV_1_ < 80% predicted

### SNPs and respiratory health outcomes

**Fig. 1:**
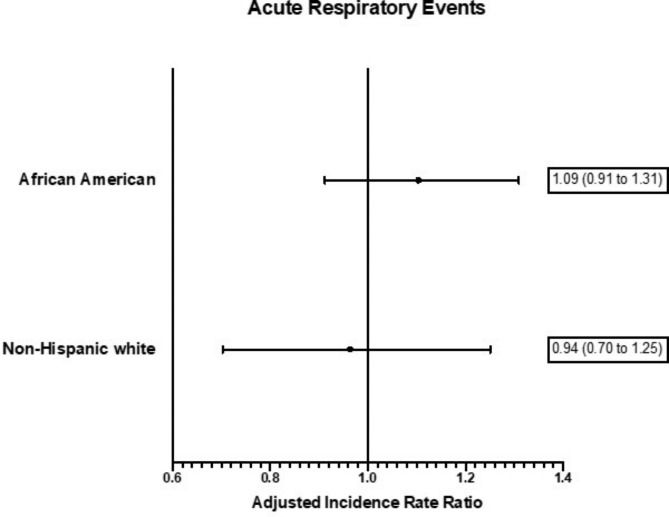
Relationship between homozygosity for rs6742078 and rate of acute respiratory events. Incidence rate ratios are adjusted for genetic ancestry, sex, age, and packs smoking at baseline and represent the ratio of rates in those homozygous for rs6742078 compared to those who have 0 or 1 copy of rs6742078

In our primary analysis, we did not find a significant relationship between the recessive (homozygous) genotype for rs6742078 and rate of acute respiratory events (Fig. 1). There were no statistically significant associations when analyses were stratified by sex (e-Table 1). Relationships between the recessive genotype for rs6742078 and secondary respiratory health outcomes are shown in Table 2. There were no significant associations in non-Hispanic white participants. In African American participants, the recessive genotype was significantly associated with a 0.08 L (95% CI -0.155 L to 0.004 L) lower baseline FVC.

**Table 2:** Relationship between recessive genotype for SNP rs6742078 and respiratory health outcomes. Estimates represent the change in the parameter of interest for the homozygosity for SNP rs6742078 vs. 0 or 1 alleles

	Non-Hispanic White		African American	
	Estimate	95% Confidence Interval	Estimate	95% Confidence Interval
Mortality - Odds Ratio	1.042	0.703 to 1.510	0.801	0.391 to 1.513
Mortality - Hazard Ratio	1.050	0.840 to 1.313	0.848	0.563 to 1.279
FEV_1_ at baseline	-0.040	-0.101 to 0.022	-0.047	-0.119 to 0.025
**FVC at baseline**	-0.015	-0.076 to 0.047	**-0.080**	**-0.155 to -0.004**
FEV1/FVC at baseline	-0.011	-0.024 to 0.001	0.003	-0.011 to 0.017
Change in FEV_1_, mL/yr	-1.650	-7.221 to 3.921	-0.939	-8.769 to 6.890
Change in FVC, mL/yr	-1.335	-9.733 to 7.062	0.515	-10.117 to 11.146
Change in Emphysema, % per yr	-0.021	-0.104 to 0.062	-0.005	-0.087 to 0.076
Change in Gas Trapping, % per yr	-0.120	-0.309 to 0.069	-0.181	-0.481 to 0.120
Change in Wall Area, mm per yr	0.011	-0.083 to 0.105	0.076	-0.100 to 0.252
Change in Pi10 mm per yr	0.002	-0.005 to 0.009	0.006	-0.007 to 0.018
Change in AWT mm per yr	0.000	-0.002 to 0.002	0.002	-0.002 -to 0.006
Change in Walking Distance, ft/yr	-20.20	-55.75 to 15.34	-10.05	-66.56 to 46.47
5-Yr Change in MMRC, Odds Ratio	1.036	0.821 to 1.298	1.327	0.986 to 1.774
5-Yr Change in Total SGRQ	-0.581	-1.988 to 0.030	1.706	-0.974 to 4.387

AWT, airway wall thickness; FEV_1_, forced expiratory volume in 1-second; FVC, forced vital capacity; MMRC, Modified Medical Research Council Dyspnea Scale; Pi10, square root of the wall area for a theoretical airway with an internal perimeter of 10 mm; SGRQ, Saint George’s Respiratory Questionnaire

Models evaluating mortality and 5-year change in mMRC as binary and survival outcomes were logistic and Cox proportional hazards models, respectively. All other regression models used linear regression. All models were adjusted for genetic ancestry, sex, age, and packs smoking at baseline

Models for all-cause mortality were additionally adjusted for clinical site at baseline

### Bilirubin intensity and respiratory health outcomes

Higher bilirubin intensity was associated with a lower risk of acute respiratory events in non-Hispanic White participants [adjusted incidence rate ratio (IRR) 0.85 per 1 unit change in bilirubin, 95% CI: 0.75 to 0.96] (Table 3). We did not analyze acute respiratory events or mortality in African American participants because we were underpowered to detect significant associations given that only 79 individuals had bilirubin intensities available. In non-Hispanic White participants higher bilirubin intensity was associated with higher baseline FEV_1_ (0.064 L per 1 unit change in bilirubin, 95% CI: 0.016 to 0.111), higher baseline FVC (0.049 L per 1 unit change in bilirubin, 95% CI: 0.001 to 0.096), and higher FEV_1_/FVC ratio (0.010 per 1 unit change in bilirubin, 95% CI: 0.001 to 0.020). There were no significant relationships between bilirubin intensities and respiratory health outcomes in African American participants.

**Table 3:** Relationship between bilirubin intensities and respiratory health outcomes. Estimates represent the change in the parameter of interest for a 1 standard deviation change in bilirubin intensity

	Non-Hispanic White		African American	
	Estimate	95% Confidence Interval	Estimate	95% Confidence Interval
**Acute Respiratory Events - IRR**	**0.85**	**0.75 to 0.96**	-	-
Mortality - Odds Ratio	0.931	0.575 to 1.419	-	-
Mortality - Hazard Ratio	0.933	0.690 to 1.264	-	-
**FEV**_**1**_ **at baseline**	**0.064**	**0.016 to 0.111**	0.044	-0.101 to 0.189
**FVC at baseline**	**0.049**	**0.001 to 0.096**	0.056	-0.110 to 0.221
**FEV1/FVC at baseline**	**0.010**	**0.001 to 0.020**	-0.003	-0.029 to 0.023
Change in FEV_1_, mL/yr	2.323	-0.998 to 5.644	-2.910	-13.864 to 8.044
Change in FVC, mL/yr	3.989	-1.358 to 9.335	3.736	-12.086 to 19.559
Change in Emphysema, % per yr	-0.019	-0.070 to 0.032	0.012	-0.096 to 0.120
Change in Gas Trapping, % per yr	-0.041	-0.135 to 9.335	0.291	-0.059 to 0.640
Change in Wall Area, mm per yr	-0.035	-0.086 to 0.032	-0.070	-0.310 to 0.169
Change in Pi10 mm per yr	-0.001	-0.004 to 0.002	-0.005	-0.019 to 0.009
Change in AWT mm per yr	-0.001	-0.002 to 0.002	-0.002	-0.008 to 0.004
Change in Walking Distance,ft/yr	8.42	-11.93 to 28.76	58.37	-17.98 to 134.71
5-Year Change in MMRC	0.026	-0.046 to 0.098	-0.074	-0.405 to 0.257
5-Year Change in Total SGRQ	-0.064	-0.892 to 0.764	0.197	-3.781 to 4.174

AWT, airway wall thickness; FEV_1_, forced expiratory volume in 1-second; FVC, forced vital capacity; MMRC, Modified Medical Research Council Dyspnea Scale; Pi10, square root of the wall area for a theoretical airway with an internal perimeter of 10 mm;SGRQ, Saint George’s Respiratory Questionnaire

Models evaluating mortality as a binary and survival outcome were logistic and Cox proportional hazards models. All other regression models used linear regression. All models were adjusted for genetic ancestry, sex, age, and packs smoking at baseline. Models for all-cause mortality were additionally adjusted for clinical site at baseline. When applicable, an offset for months followed was used

No mortality events occurred for African-Americans for this subset of data

## Discussion

In this cohort of current and former smokers, we confirmed previous findings of ours and others that higher bilirubin levels are associated with fewer acute respiratory events, but our novel analysis of a genetic determinant of higher bilirubin was not associated with acute respiratory event risk. Our analysis suggests that the association between higher bilirubin levels and decreased AECOPD may be due to confounding, and that interventions directed towards increasing bilirubin levels are not likely to improve respiratory health outcomes.

Ample evidence supports the idea that increasing bilirubin levels might lead to a decreased risk of acute respiratory events. Higher serum bilirubin has been associated with a decreased risk of AECOPD in two previous observational analyses. [8,9] Brown and colleagues found that a higher serum bilirubin level was associated with a decreased risk of AECOPD in a secondary analysis of two randomized controlled trials (n = 853 in development cohort and n = 1018 in validation cohort; validation cohort adjusted hazard ratio 0.80 per log_10_ increase, 95% CI: 0.67 to 0.94). [8] Leem and colleagues similarly found that higher serum bilirubin was associated with a decreased risk of AECOPD (n = 535).^9^ Bilirubin is hypothesized to have beneficial effects in COPD through antioxidant properties, and there are basic and translational science data implicating oxidative stress in COPD and COPD exacerbations. [24] Additionally, use of medications with antioxidant activity may reduce the frequency of COPD exacerbations, but we are unaware of any studies that have targeted bilirubin levels as a therapeutic intervention in COPD. [25,26] These previous observational studies seemed to support increased bilirubin levels as a potential therapeutic target in COPD, but observational data are prone to confounding and reverse causality, and promising basic and translational science data do not always translate to effective clinical interventions. We had contemplated pursuing intervention studies using existing drugs such as atazanavir, a protease inhibitor that inhibits UGT1A1 and has been used to increase bilirubin levels in diabetics [27], but given known genetic associations with bilirubin levels, we elected to pursue this analysis before expending the considerable resources required for a clinical trial. Genetic analyses are one approach to overcome some of the barriers of observational data.

We utilized a Mendelian randomization approach to test the association between homozygosity for SNP rs6742078 and the risk of acute respiratory events. Every individual inherits one of two versions of a gene and Mendelian randomization leverages this random allocation to test the causal effect of a trait (e.g. higher serum bilirubin levels) on an outcome (e.g. lower rate of acute respiratory events as long as three criteria are met [14]:

1. There is a genetic change (in our study homozygosity for rs6742078) associated with the trait of interest (higher bilirubin intensities). Though we did not have serum bilirubin concentrations in COPDGene, we utilized bilirubin intensities from mass spectrometry to show that homozygosity for the SNP rs6742078 was associated with higher bilirubin levels. This is consistent with previous studies, including a GWAS. [16,18].
2. There is no association between the genetic change (homozygosity for rs6742078) and the outcome of interest (acute respiratory events) except through the trait (higher serum bilirubin levels). This criterion is difficult to prove definitively, but rs6742078 has no known effects other than to increase bilirubin through UGT1A1 activity, and other than through increased bilirubin levels there is no other known pathway by which this genetic change would influence risk of acute respiratory events.
3. There are no confounding factors in the relationship between the genetic change (homozygosity for rs6742078) and the outcome of interest (acute respiratory events). Similar to criteria 2), this is difficult to prove definitively, but unlike SNPs in some promoter regions, rs6742078 does not have known pleiotropic effects that would confound the relationship between bilirubin levels and acute respiratory events. [14].

We did find that higher bilirubin intensity was associated with a decreased rate of acute respiratory events, providing further observational evidence that higher bilirubin levels are associated with a decreased risk of acute respiratory events/AECOPD.^8,9^ The combination of these observational findings with the negative Mendelian randomization analysis suggest that residual confounding or reverse causality may account for the associations between bilirubin levels and COPD outcomes. Residual confounding could result from underlying physiology, nutritional status, co-morbidity burden, socioeconomic factors, or medications. Reverse causality could lead to these disparate findings through an inflammatory process in the lungs leading to susceptibility to acute respiratory events as well as lower serum bilirubin levels through consumption of bilirubin in the process of scavenging free oxygen radicals or changes in bilirubin metabolism driven by inflammation.

Our findings differed from those of Curjuric and colleagues who analyzed 4195 subjects from a Swiss cohort and found that homozygosity for rs6742078 was associated with a higher cross-sectional FEV_1_/FVC ratio. [18] They did not find associations with FEV_1_ or FVC on their own, and did not analyze respiratory events or longitudinal changes. One possible reason for these differing findings is that approximately 33% of participants in that study were never smokers (versus 0% in our COPDGene study), and the relationship between bilirubin levels and lung function may differ by smoking status. [28] Dai and colleagues recently published an analysis showing a non-linear association between total bilirubin and COPD (defined as post-bronchodilator FEV_1_/FVC ratio < 0.7), where lower and higher levels of bilirubin were both associated with increased risk. In that analysis, they also performed a Mendelian randomization study, and similar to our analysis did not find any association between genetic determinants of bilirubin levels and COPD. [29].

We did find one statistically significant association between homozygosity for rs6742078 and secondary respiratory outcomes in African American participants. Homozygosity for rs6742078 was associated with lower (worse) FVC at baseline. We did not adjust for multiple testing, so these findings might represent type I statistical error, but larger studies in those of African ancestry would be required to better address this finding.

Our study has some important limitations. COPDGene is a cohort of only current and former smokers, and at least one previous analysis found that the relationship between bilirubin and respiratory health may be stronger in never smokers. [28] We also did not have clinical serum bilirubin levels available and used bilirubin intensities from mass spectrometry to test the association between SNPs and bilirubin levels, and between bilirubin levels and respiratory health outcomes. Though bilirubin intensities from this mass spectrometry method had only moderate correlation with measured bilirubin concentrations in a previous study (Spearman correlation 0.66) [30], this would not influence our primary goal of testing associations between genetic determinants of bilirubin levels and respiratory health outcomes. We also chose to analyze acute respiratory events rather than AECOPD to maximize power. Though the previous analyses that led to this work were primarily in people with COPD, smokers without COPD by spirometry are known to have clinically important respiratory events. [31,32] Finally, though our results are negative, our 95% confidence intervals could not exclude up to a 30% reduction in the rate of acute respiratory events in those with the recessive genotype.

Our study also has several strengths. The use of the COPDGene cohort allowed us to test the association between a SNP that is robustly associated with bilirubin levels and respiratory health outcomes in over 8000 well-characterized persons with up to 5 years of follow-up. This is the first study we are aware of to test the relationship between a genetic determinant of bilirubin levels and acute respiratory events or AECOPD. The longitudinal design also allowed us to analyze longitudinal lung function decline and other markers of respiratory health.

## Conclusion

A genetic determinant of bilirubin levels was not associated with acute respiratory event risk in this Mendelian randomization study. Our results suggest that the association between higher bilirubin levels and better respiratory health is not causal and do not support targeting higher bilirubin levels as a therapeutic target for preventing acute respiratory events. These results require replication in other cohorts.

## Electronic supplementary material

Below is the link to the electronic supplementary material.

Supplementary Material 1

## Data Availability

COPDGene datasets are publicly available (dbGaP accession number phs000179.v1.p1).

## Declarations

### Disclaimer

The views expressed in this article are those of the authors and do not reflect the views of the United States Government, the Department of Veterans Affairs, the National Institutes of Health, the funders, the sponsors, or any of the author’s affiliated academic institution.
