# Supplementary material for: Bilirubin-associated single nucleotide polymorphism (SNP) and respiratory health outcomes: a mendelian randomization study
Source: Respir Res. 2023 Jul 20;24:190. doi: 10.1186/s12931-023-02471-w (PMC10357606; doi:10.1186/s12931-023-02471-w)
Supplement: Supplementary file 1 — Supplementary Material 1 [file 12931_2023_2471_MOESM1_ESM.docx]

**Supplementary Materials**

**Association between bilirubin-associated single nucleotide polymorphism (SNP) and respiratory health outcomes**

*Arianne K. Baldomero^1,2^, *David M. MacDonald^1,2^, Adam Kaplan^3^, Eric Lock^3^, Michael H. Cho^4^, Russell Bowler^5^, Lucas Gillenwater^5^, Ken M. Kunisaki^1,2^, and Chris H. Wendt^1,2^

* These authors contributed equally to this work.

1: Pulmonary, Allergy, Critical Care, and Sleep Medicine, Minneapolis Veterans Affairs Health Care System, Minneapolis, MN, USA

2: Pulmonary, Allergy, Critical Care, and Sleep Medicine, University of Minnesota, Minneapolis, MN, USA

3: Department of Biostatistics, University of Minnesota, Minneapolis, MN, USA

4: Division of Pulmonary and Critical Care, Department of Medicine, Brigham and Women's Hospital, Harvard Medical School, Boston, MA, USA

5: Department of Medicine, National Jewish Health, Denver, CO, USA

**Contents:**

**e-Figure 1**: Histogram of bilirubin intensities.

**e-Figure 2**: Box plots for bilirubin levels by SNP count for the SNPs of interest.

**e-Figure 3**: Correlation grid for SNPs of interest.

**e-Table 1**: Associations between SNP of interest and rate of acute respiratory events, stratified by sex.


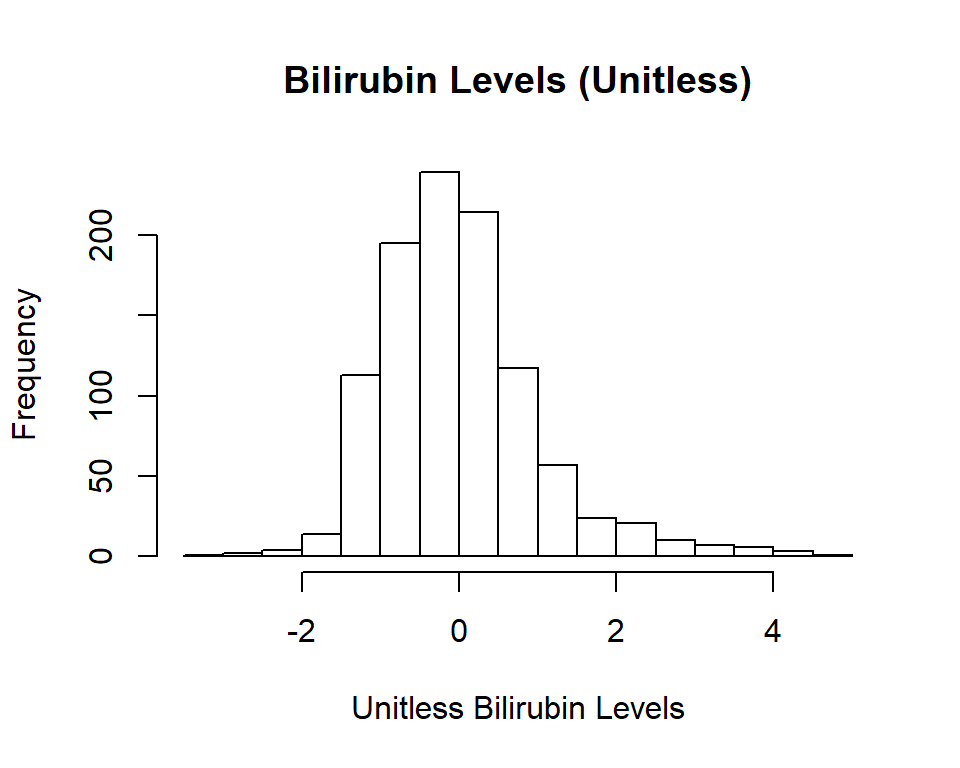


**e-Figure 1**: Histogram of unitless bilirubin levels. Bilirubin intensities were obtained from mass spectrometer for 1,078 participants. The bilirubin intensities were centered and scaled to have mean 0 and standard deviation 1.


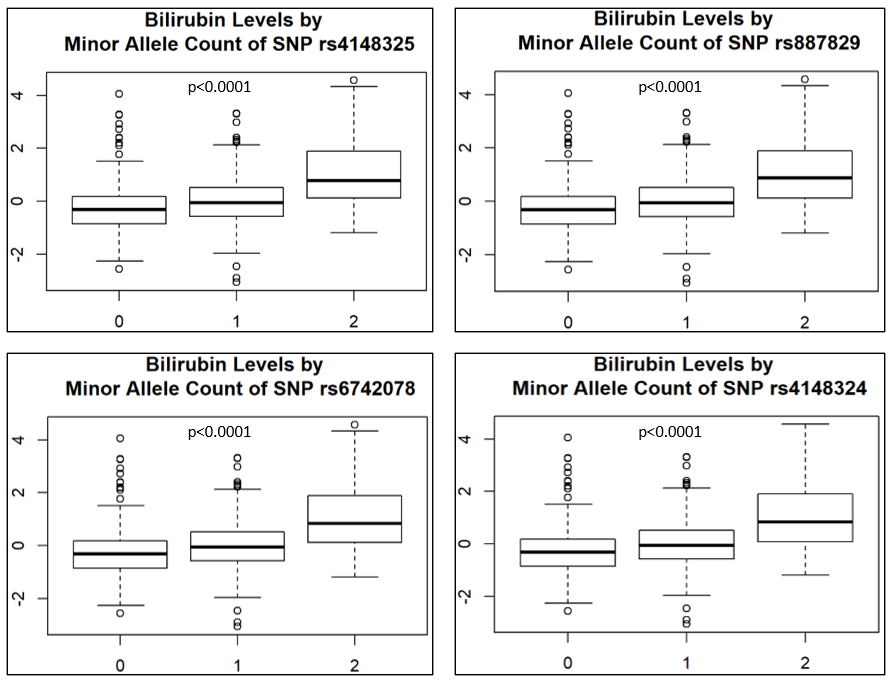


**e-Figure 2:** Unitless bilirubin levels for 0, 1, or 2 copies of the SNPs of interest. All four SNPs showed similar associations, where there was no difference in bilirubin levels between 0 and 1 copy, but bilirubin levels in participants with 2 copies were significantly higher than either those with 0 or 1 copy. p-values are from ANOVA F-tests.


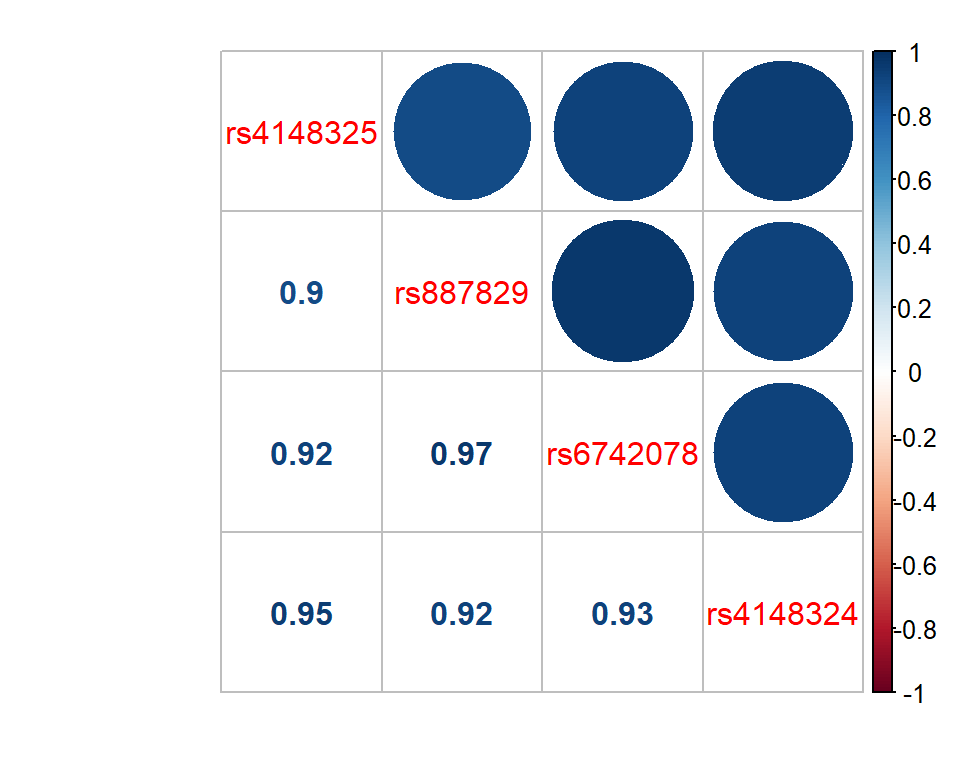


**e-Figure 3:** Correlation plot for the SNPs of interest. Correlations were tested using Pearson’s Correlation Test and all were > 0.9. Based upon these results and previous literature we performed further analyses using only SNP rs6742078.

**e-Table 1**: Associations between recessive phenotype (homozygous for SNP rs6740278) and rate of acute respiratory event, stratified by sex. There were no significant associations in males or females in either non-Hispanic white or African American participants, and the credible intervals overlap, indicating that relationships were not significantly different between males and females.

|  | Effect (median) | 95% credible interval |
| --- | --- | --- |
| Non-Hispanic white | | |
| Female | 1.03 | 0.93 to 1.14 |
| Male | 1.06 | 0.93 to 1.21 |
| African American | | |
| Female | 1.09 | 0.91 to 1.30 |
| Male | 0.90 | 0.68 to 1.16 |
